# MicroRNA Regulation in Infectious Diseases and Its Potential as a Biosensor in Future Aquaculture Industry: A Review

**DOI:** 10.3390/molecules28114357

**Published:** 2023-05-26

**Authors:** Aileen See SiouNing, Tang Swee Seong, Hidehiro Kondo, Subha Bhassu

**Affiliations:** 1 Animal Genomic and Genetics Evolutionary Laboratory, Department of Genetics and Microbiology, Institute of Biological Sciences, Faculty of Science, University of Malaya, Kuala Lumpur 50603, Malaysia; 2 Terra Aqua Laboratory, Centre for Research in Biotechnology for Agriculture (CEBAR), Research Management and Innovation Complex, University of Malaya, Kuala Lumpur 50603, Malaysia; 3 Microbial Biochemistry Laboratory, Division of Microbiology and Molecular Genetic, Institute of Biological Sciences, Faculty of Science, University of Malaya, Kuala Lumpur 50603, Malaysia; 4 Laboratory of Genome Science, Tokyo University of Marine Science and Technology, Tokyo 108-8477, Japan

**Keywords:** microRNA, infectious diseases, biomarker, therapeutics, biosensor, aquaculture

## Abstract

An infectious disease is the most apprehensive problem in aquaculture as it can lead to high mortality in aquatic organisms and massive economic loss. Even though significant progress has been accomplished in therapeutic, prevention, and diagnostic using several potential technologies, more robust inventions and breakthroughs should be achieved to control the spread of infectious diseases. MicroRNA (miRNA) is an endogenous small non-coding RNA that post-transcriptionally regulates the protein-coding genes. It involves various biological regulatory mechanisms in organisms such as cell differentiation, proliferation, immune responses, development, apoptosis, and others. Furthermore, an miRNA also acts as a mediator to either regulate host responses or enhance the replication of diseases during infection. Therefore, the emergence of miRNAs could be potential candidates for the establishment of diagnostic tools for numerous infectious diseases. Interestingly, studies have revealed that miRNAs can be used as biomarkers and biosensors to detect diseases, and can also be used to design vaccines to attenuate pathogens. This review provides an overview of miRNA biogenesis and specifically focuses on its regulation during infection in aquatic organisms, especially on the host immune responses and how miRNAs enhance the replication of pathogens in the organism. In addition to that, we explored the potential applications, including diagnostic methods and treatments, that can be employed in the aquaculture industry.

## 1. Introduction

Aquaculture is the process of culturing, breeding, and harvesting aquatic organisms, which is an alternative way of producing seafood for human consumption. Seafood is the highest protein retention compared to poultry, pork, and beef, which indicates that seafood is a high protein source for human consumption [1]. However, the ever-growing human population could increase the demand for seafood. Unfortunately, overcapturing wild aquatic organisms or overfishing of oceans cannot fill up the gap in the seafood supply. Therefore, aquaculture plays an important role in providing healthy and environmentally friendly seafood production. According to FAO, 2022, aquaculture production expanded in the late 1980s, but it was growing slowly from 2018 to 2020 [2]. These lower growth rates may have occurred due to several factors, including the rise of diseases, changes in policy, and the COVID-19 pandemic that impacted marketing and led to a reduced workforce and restricted exportation. Nevertheless, diseases remain an issue and a challenge in aquaculture. 

Aquatic animals are susceptible to bacterial, viral, fungal, and parasitic infection and could reduce aquaculture production, which directly leads to massive economic losses. For example, vibriosis bacterial diseases, infectious haematopoietic necrosis diseases, white spot diseases, and others. In addition, there are emerging and re-emerging viruses in aquaculture including *Lymphocystis* disease virus, infectious haematopoietic necrosis virus, infectious salmon anaemia virus, piscine orthoreovirus, Tilapia Lake virus, covert mortality nodavirus, shrimp hemocyte iridescent virus, and abalone herpesvirus [3]. Therefore, surveillance and laboratory diagnosis should be practiced routinely in aquaculture to prevent the outbreak of diseases. Moreover, various prevention measures have been developed to control the diseases in aquaculture, including vaccines, antibiotics, and probiotics. Watanabe and Sakami (2021) mentioned that the use of vaccines has reduced diseases from about 10 to 4% of the total production since 2000 [4]. However, vaccination in invertebrates is problematic as they lack immunological memory to defend against diseases. For example, the white spot syndrome virus (WSSV) is the most devasting viral infection in crustacean aquaculture as it can cause rapid outbreaks and high mortality. To make the aquaculture industry more sustainable and profitable, advanced technologies should be developed to control aquatic diseases. 

Over the last two decades, genome information of organisms has been increasingly revealed through genome sequencing technologies. There is another method to analyse the transcriptomic data, which is RNA-seq technology. RNA-seq technology is beneficial in understanding gene expression, gene regulatory networks, metabolic pathways, and protein–protein interaction [5]. Thus, this enables various biological processes to be revealed, such as immune responses and development, stress responses, etc. In addition to that, RNA-seq technology can also identify the relationship between messenger RNA (mRNA) and non-coding RNAs of organisms, such as microRNAs (miRNAs) and long non-coding RNAs (lncRNAs), under various conditions.

lncRNAs are RNA molecules with more than 200 nucleotides (nt) without protein-coding ability. lncRNAs participate in various biological processes, such as reproduction, immune responses and development, and others, by regulating gene transcriptional, post-transcriptional, and epigenetic levels [6,7]. There are four action modes in lncRNAs mechanism to interact with a range of DNAs, RNAs, and proteins to modulate the biological functions: (1) acts as molecular signalling inducer that can be used alone or interact with other transcriptional factors or enzymes to mediate the transcription; (2) acts as a molecular decoy which binds to functional proteins or miRNAs, thereby blocking the gene regulation or inhibitory effect of miRNAs on mRNA; (3) acts as a molecular guide to carry the functional proteins and localize on the target DNA area to perform the function; (4) acts as a molecular scaffold in which the same lncRNA binds to multiple transcription factors to regulate gene transcription [8,9,10]. Among these, the interaction between lncRNAs and miRNAs is the predominant action mode found in aquaculture that has been revealed by several studies [11,12,13,14]. However, lncRNAs are less genetically conserved across the species compared to miRNAs, but they can regulate most levels of gene regulations [15]. Nevertheless, miRNAs are the most abundantly studied in different organisms. miRNAs are the non-coding RNA molecules that are used to control the number of proteins that are translated from the mRNAs. Hence, miRNAs are used to control gene expression and directly affect its biological processes. Several studies revealed the transcriptomic data of miRNAs from aquatic organisms under environmental stress and infectious diseases using high-throughput sequencing technologies [16,17,18,19,20]. These studies have suggested that the identified miRNAs could serve as potential biomarkers for disease prevention and treatment.

miRNAs are the small non-coding RNAs transcribed from DNA by RNA polymerase II with approximately 20 to 22 nt in length. They act as post-transcriptional regulators to mediate gene expression by degrading or destabilizing the messenger RNAs (mRNAs) and inhibiting protein translation. However, some miRNAs could interact with 5′ untranslated region (5′UTR) of genes, promoters, or coding regions to activate the gene expression under certain environmental conditions [21,22]. In 1993, the first miRNA lin-4 in *Caenorhabditis elegans* was found by Ambros and Ruvkun’s groups, which repressed lin-14 mRNA for progression from the first to the second larval stage, but produced two non-coding RNAs with 21 and 61nt in *C. elegans* [23,24]. After that, the same groups along with Wightman found a small RNA that had antisense complementarity to multiple sites in the 3′untranslated region (3′UTR) of lin-14 mRNA and repressed its protein expression without causing significant changes in mRNA level [24]. Starting in the year 2000, homologous miRNAs have been discovered in various animal organisms, including humans, using high-throughput sequencing technology [25,26,27]. Furthermore, the functions of these recognized miRNAs were predicted by bioinformatics tools. This resulted in a dedicated miRNA database being established called miRBase 21, which serves as fundamental information for family classification, annotation, and function of discovered miRNAs.

## 2. MicroRNA Biogenesis and General Mechanism

**Figure 1:**
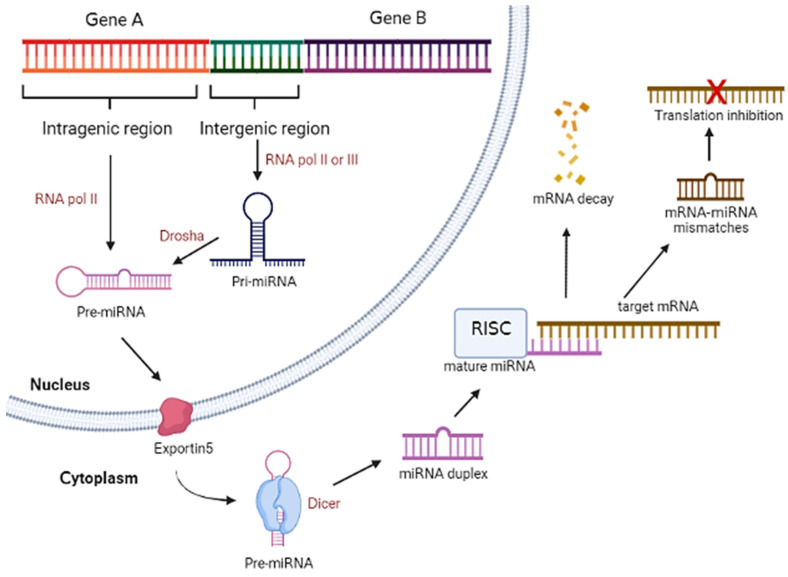
Overview illustration of miRNA biogenesis in animals. (Created with Biorender.com).

MiRNAs are derived from 60–70 nt of double-stranded RNA hairpin precursors and these precursors are normally found in clusters within the intergenic or intragenic regions of the host genome. Intergenic miRNAs are transcribed from the miRNA genes by their own promoters that are located in between protein-coding genes, while intragenic miRNAs are generated from the protein-coding genes by the same promoters [28]. Intergenic miRNAs are transcribed by RNA polymerase II or III and form large stem-loop primary miRNAs (pri-miRNAs) and are further processed into shorter hairpin pre-mature miRNAs (pre-miRNA) by a protein complex called microprocessor which contain RNase III endonuclease Drosha and RNA binding protein DiGeorge syndrome critical region 8 (DGCR8). In contrast, intragenic miRNAs are transcribed from introns of protein-coding genes by RNA polymerase II to form pre-miRNAs. The formed pre-miRNAs are further sliced by spliceosomal complex or microprocessor to form pre-miRNAs that could be exported into the cytoplasm by nucleocytoplasmic factor exportin 5 or RanGTP. The exported pre-miRNAs are then processed by Dicer to form miRNA duplex or by Ago2 to generate Ago2-cleaved precursor miRNA (ac-pre-miRNA) that can be a substrate for Dicer [29]. The mature miRNAs assemble with Argonaute protein to form an RNA-induced silencing complex (RISC), and the miRNAs unwind into a single guide strand while another strand known as the passenger strand will be removed from the complex. Thus, the complex can bind to the mRNA target to induce mRNA degradation by perfect matching, mRNA destabilization, and deadenylation by some mismatches, leading to translation inhibition [28]. Figure 1 shows the overview illustration of miRNA biogenesis in organism.

## 3. MicroRNA Regulation in Aquaculture Responses to Infectious Diseases

The mechanism of miRNA is relatively similar to small interfering RNA (siRNA). However, they have the following differences: siRNA is exogenous RNA while miRNA is endogenous RNA which is encoded by the hosts’ genome; siRNA is used to silence the target mRNA while miRNA is used to either regulate or inhibit the expression of multiple mRNAs. Therefore, miRNAs could be used to upregulate or downregulate the mRNAs in host organisms to respond to various conditions. Recently, many studies have revealed through transcriptomic analysis that various miRNAs are significantly expressed in aquatic organisms in response to infectious diseases. [30,31,32,33,34]. For example, miRNAs regulations have been revealed in pathogenic protozoan parasites Ectoparasitic dinoflagellate *Amyloodinium ocellatum* infected golden pompano (*Trachinotus ovatus*) [30]. The infectious diseases could affect the miRNAs expression to inhibit or facilitate the infection by targeting the hosts’ mRNAs. The following section will discuss the responses that could be induced by miRNA expression in response to infectious diseases in aquatic organisms.

### 3.1. Innate Immunity

Innate immunity is the first line of non-specific defense against pathogens entering the host body. Innate immunity is initiated by pathogen-associated molecular patterns (PAMPs) that could be recognized by pattern recognition receptors (PRRs) and subsequently trigger both the cellular and humoral immune components in a complex manner, including immune recognition, signal transduction and effector production [35]. Cellular innate immunity provides the physical barrier in the form of mucus and epithelial cells in the outer protective layers, such as skin, shell, gills, and stomach, against the pathogens by triggering various immune responses, such as phagocytosis, encapsulation, nodulation formation, and apoptosis, to eliminate the pathogens [36,37]. Whereas, humoral immunity utilises various proteins and glycoproteins, such as antimicrobial peptides (AMPs), clotting proteins, agglutinins, proteinase inhibitors, and so forth, to destroy or inhibit the pathogens by inducing the first line of defense mechanisms. For instance, prophenoloxidase cascade, clotting cascade, induction of lectins, and the cytoplasmic signal transduction cascades include Toll and immune deficiency (IMD) pathways, and others [37,38]. Nevertheless, all these immune responses could be regulated by various miRNAs during infections. The miRNA is a double-edged sword, which could bring either positive or negative effects to the immune system.

#### 3.1.1. Autophagy

The findings by Zhou’s group suggested that the miRNAs could regulate autophagy during infectious spleen and kidney necrosis virus (ISKNV) infection in mandarin fish [32]. Autophagy is the natural self-degradation of damaged cells or dysfunctional components via a lysosome-dependent mechanism. Thus, autophagy plays an important role in the response to pathogen infection. Liu et al. (2015) found that the autophagy activity was upregulated during spring viraemia of carp virus (SVCV) infection and improved the survival rate of infected cells [39]. On the other hand, Zhou et al. (2022) revealed that several miRNAs could regulate autophagy-related genes (Atg) and affect the autophagy activity in ISKNV-infected mandarin fish [32]. For example, novel_miR-61 targets Atg7, and novel_miR-205 and novel_miR-320 target Atg16 genes are upregulated in ISKNV-infected mandarin fish at an early stage (12 and 24 hours post-infection).

#### 3.1.2. Toll and IMD Pathways

Interestingly, Ou et al. (2022) investigated the immunity and autophagy in *Macrobrachium nipponense* shrimp hemocytes infected with *Spiroplasma eriocheiris* bacteria through integrated analysis of mRNA and miRNA transcriptomes [13]. They found that 25 miRNAs were involved in Toll and IMD pathways, 41 in endocytosis, 30 in the lysosome, and 12 in the vascular endothelial growth factor (VEGF) pathway. Toll and IMD pathways are initiated when the PAMPs of pathogens are recognized by specific PRRs and further activate a cascade that could induce antimicrobial peptides (AMPs) to regulate innate immunity. In the study of Ou et al. (2022), serine protease 4 was found to be regulated by the newly discovered miRNA called PC-5p-707_5272 [33]. In the Toll signalling pathway, recognition of PAMPs by PRRs leads to the activation of the serine protease cascade, which could cause the transformation of prophenoloxidase into phenoloxidase that acts as an enzyme to catalyse melanin production [40]. Other than that, they also found that another three genes belonging to Toll and IMD pathways, Relish, Dorsal, and NF-κB inhibitor α, were upregulated in *S. eriocheiris*-infected hemocytes of *M. nipponense*. In addition, multiple miRNAs that target these genes were revealed in their study, such as dpu-miR-1 and PN-isc-miR-315_R+2, etc. targeted Relish, dpu-miR-317_R-2, bmo-miR-2a-3p_R+2_1, etc. targeted Dorsal, while pte-miR-315-5p_R-3, PC-5p-6595_648, etc. targeted NF-κB inhibitor α. However, these miRNAs were not all downregulated, which might be due to the more complex regulatory networks affecting the mRNA expression.

**Figure 2:**
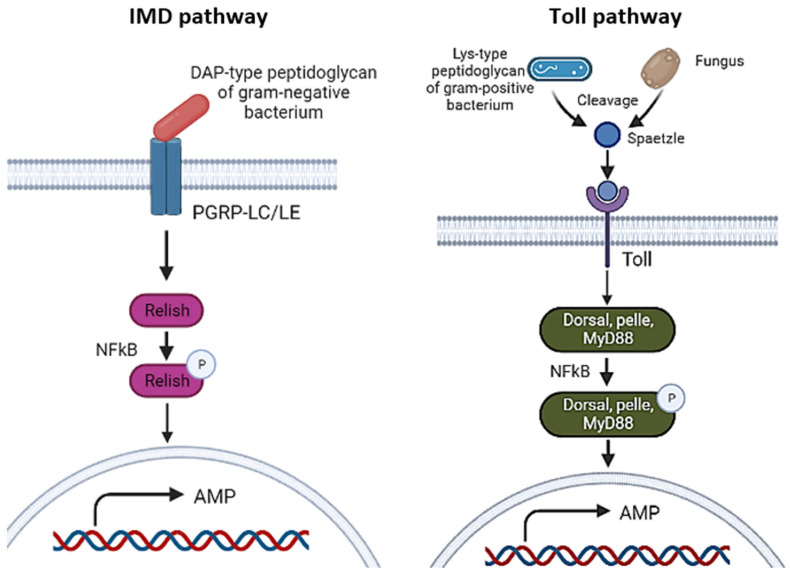
Schematic diagram of comparisons between IMD and Toll pathways (Created with Biorender.com).

Activation of the IMD and Toll pathways could trigger the NF-κB (nuclear factor kappa B) signalling pathway, which involves protein complexes that control DNA transcription and regulate different processes of immune responses, inflammatory responses, cell proliferation, and apoptosis (see Figure 2). The IMD pathway is mainly activated by Gram-negative bacterial infection through the recognition of Gram-negative bacterium-derived diaminopimelic acid (DAP)-type peptidoglycan by the receptors peptidoglycan recognition proteins LC (PGRP-LC) and -LE. This recognition subsequently induces different kinase regulatory complexes to phosphorylate and cleave the NF-κB-related transcription factor Relish into N-terminal and C-terminal Relish fragments, which can be translocated into the nucleus to trigger AMP gene transcription [41]. In contrast, the Toll pathway is activated by Gram-positive bacterial and fungal infections through the recognition of Gram-positive bacterium derived by receptors PGRP-SA and -SD, as well as the detection of glucans (polymers of D-glucose) present in the fungal cell wall by glucan-binding protein 3 (GNBP3) [41,42]. Both infections subsequently lead to the activation of the protease cascade to cleave the host protein, Spaetzle. This cleaved Spaetzle serves as a ligand for Toll receptors and induces Toll signalling in cytoplasmic components, such as Dorsal, Pelle, MyD88, Cactus, and Dorsal-related immune factor (DIF), to trigger the AMP expression in the nucleus [41]. Furthermore, the NF-κB inhibitor α (IκBs) inhibits the NF-κB signalling pathway by binding to the NF-κB regulatory protein to form an inactive trimer in the cytoplasm, which enables this inactive trimer to translocate into the nucleus to induce transcription. As mentioned above, the NF-κB signalling pathway is activated to induce AMPs and defeat pathogens during infection. Hence, the IκBs are degraded when exposed to cytokines or microbial products, decreasing their inhibitory effect on the NF-κB pathway [43]. Pazarentzos et al. (2014) found that IκBs exert apoptotic activity as they can inhibit the anti-apoptotic NF-κB protein [44]. They revealed that the IκB protein is localized in the outer mitochondrial membrane and interacts with voltage-dependent anion channel and mitochondrial hexokinase II, preventing the release of Bax-mediated cytochrome c for apoptosis purposes. Thus, the NF-κB signalling pathway not only induces an immune response to kill pathogenic microorganisms but also triggers cell death resistance [45].

#### 3.1.3. Vascular Endothelial Growth Factor (VEGF) Pathway

On the other hand, there are 12 miRNAs revealed in the miRNA transcriptomic study by Ou et al. (2022) [33]. For example, putative leupaxin isoform X1 was regulated by dpu-miR-317_R-2; PC-5p-1243_3142 miRNAs and paxillin-like transcripts were regulated by dpu-miR-8_R-1; PC-5p-1243_3142 miRNAs. VEGF pathway plays an important role in angiogenesis, vascular permeability, cell migration in macrophage lineage and endothelial cells, and cell proliferation [46]. Interestingly, the VEGF pathway is also involved in the host organisms during pathogenic infections. The study found that the transcription levels of genes involved in the VEGF pathway were upregulated in shrimp *Fenneropenaeus chinensis* response to WSSV infection [47]. Therefore, the researchers subsequently studied the role of VEGF genes in viral infection. Several VEGF genes (*Lv*VEGF1, *Lv*VEGF2, *Lv*VEGF3, *Lv*VEGF4, and *Lv*VEGF5) were reported in *Litopenaeus vannamei* under WSSV infection. In *L. vannamei*, VEGF genes have been found to be responsible for WSSV replication, and silencing these genes using the RNAi method could reduce the in vivo copy number of WSSV and shrimp mortality [48,49,50,51,52]. In addition to that, they also investigated the knockdown of *Lv*VEGF4 and *Lv*VEGF5 genes which could repress the expression of focal adhesion tyrosine kinase (FAK) and phosphoinositide 3-kinase (PI3K) genes, which act as regulators for cell migration and VEGF-inhibited apoptosis, respectively, in VEGF pathway [52]. In addition, the transcriptomic analysis also showed the expression of VEGF receptor 2-like transcript was upregulated in WSSV-infected *L. vannamei* shrimps for 2 h post-infection [53]. In mammalian, the receptor VEGF-2 plays a crucial role in angiogenesis which could induce various signalling pathways, including phospholipase-Cγ (PLC-γ)/protein kinase C (PKC), p38-Mitogen-activated protein kinase (MAPK), PI3K/protein kinase B (PKB), SRC, and FAK to trigger processes such as cell migration, adhesion, proliferation, survival, and permeability to the response of cancer [54]. Even though studies have suggested that it enhanced WSSV replication in shrimp *L. vannamei*, it has been reported that it could also regulate immune response by inducing the expression of AMPs. For instance, the p38/MAPK pathway initiated intestinal innate immunity in *Caenorhabditis elegans* under *Pseudomonas aeruginosa* infection [55]. In addition to that, Inada et al. (2013) reported that *Mj*VEGF and *Mj*VEGF2 genes promoted innate immunity in Kuruma shrimp *Marsupenaeus japonicus* [56].

#### 3.1.4. Janus Kinase-Signal Transducer and Activator of Transcription (JAK/STAT) Signalling Pathway

**Figure 3:**
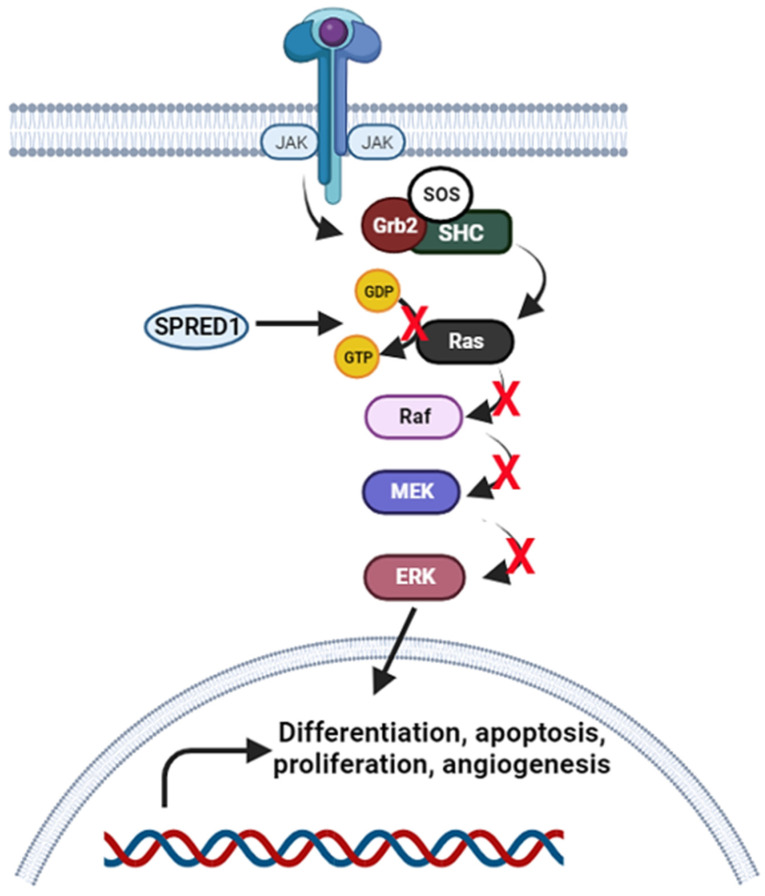
Schematic diagram of inhibition of Ras/Raf/ERK signalling pathway by SPRED1. (Created with Biorender.com).

A wide range of cytokines and growth factors are essential in inducing JAK/STAT signalling pathway, leading to crucial cellular responses such as cell proliferation, migration, differentiation, apoptosis, and immunity. This pathway is initiated when JAKs are activated by cytokine stimulation and cause the phosphorylation of STATs to form STAT dimers. These dimers are then subsequently translocated into the nucleus to regulate the downstream target gene expression. According to Ou et al. (2021), four miRNAs related to JAK/STAT signalling pathway were significantly expressed in *Spiroplasma eriocheiris*-infected *M. rosenbergii* including PC-mro-3p-103, PC-mro-5p-62, PN-mro-miR-306, and PN-mro-miR-316 [57]. Among these miRNAs, the target of PC-mro-3p-103, PN-mro-miR-306, and PN-mro-miR-316 are sprouty-related EVH1 domain containing 1 (SPRED1), while PC-mro-5p-62 is a protein inhibitor of activated STAT. SPRED1 is an inhibitor of receptor tyrosine kinase (RTK)-dependent Ras/Raf/ERK signalling pathway by suppressing Ras and Raf components. The Ras/Raf/ERK signalling pathway is a central signal transduction pathway that is related to JAK/STAT pathway. It is initiated when interleukin 3 (IL-3) binds to the IL-3 receptor and activates the JAK/STAT associated coupling complex of SHC/Grb2/SOS. This leads to inactive Ras GDP (guanosine diphosphate) protein becoming an active conformation of Ras GTP (guanosine triphosphate) protein. This phosphorylation enhances Raf activity that could activate MEK and phosphorylates ERK to trigger downstream events such as cell differentiation, proliferation, angiogenesis, and prevention of apoptosis [58,59,60] (see Figure 3). Thus, when SPRED1 suppresses Ras and Raf activity, it directly inhibits all these cellular responses. In addition to that, SPRED also negatively regulates the VEGF pathway that is related to the angiogenesis process. Wang et al. (2008) reported miR-126 increased angiogenesis activity through the VEGF pathway when silencing the SPRED1 gene in mice [61].

### 3.2. Oxidative Phosphorylation Pathway

**Figure 4:**
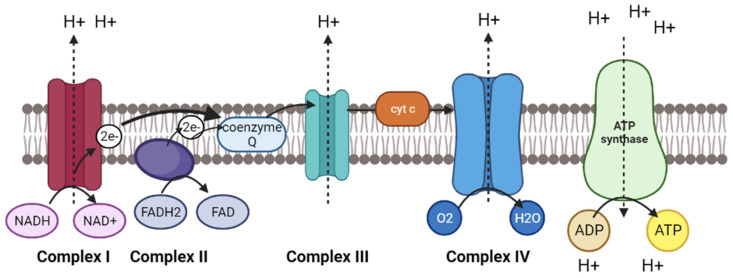
Schematic representation of oxidative phosphorylation pathway in mitochondrial to generate ATP energy (Created with Biorender.com).

The oxidative phosphorylation pathway is derived from the electron transport chain reaction and chemiosmosis process to generate energy in the form of adenosine triphosphate (ATP). It uses a series of proteins and organic molecules to transfer the electrons and protons across the mitochondria through an electrochemical transmembrane gradient from a higher to a lower energy level. Figure 4 shows the schematic representation of oxidative phosphorylation pathway in mitochondrial. Firstly, the electrons are released through the reduction of NADH and FADH_2_ molecules and transferred into complex I and II, respectively. Complex I uses the free energy derived from released electrons to pump protons from the mitochondrial matrix to intermembrane space. The electrons are then transferred to a small mobile electron carrier called coenzyme Q, and deliver the electrons through the membrane to complex III. As more electrons are transferred from coenzyme Q to complex III, more protons are pumped across the membrane through complex III. Next, the electrons are delivered to a peripheral protein called cytochrome c, which carries the electrons to complex IV, where the final protein pumps the protons across the membrane. In complex IV, the electrons separate the oxygen molecule into two oxygen atoms and combine them with two protons to form water. Thus, four electrons are required to split one oxygen molecule and accept four protons to form two water molecules. In the end, the protons flow through the complex V and ATP synthase from a higher concentration of protons in an intermembrane space to a lower concentration of mitochondrial matrix. This proton gradient is used to catalyse the ATP synthase to add phosphate ions to ADP and form an ATP molecule. This process is also known as chemiosmosis.

Therefore, the oxidative phosphorylation pathway is important because it provides ATP as an energy source in living organisms to support their life and maintain metabolic processes, including anabolic and catabolic processes. The anabolic process involves a synthesis of compounds such as carbohydrates, proteins, lipids, and nucleic acids through polymerization, while the catabolic process is a pathway of breaking down the compounds through cellular respiration, such as glycolysis, Krebs cycle, urea cycle, fatty acid oxidation, and other oxidation processes. As the oxidative phosphorylation pathway is vital in energy metabolism, damage to one or more proteins and complexes required in the electron transport chain would deplete the ATP synthesis and subsequently affect cellular respiration [62].

Several studies revealed that various miRNAs could affect the regulation of transcripts associated with the oxidative phosphorylation pathway in virus-infected shrimps [63,64,65]. Tan et al. (2013) found several novel miRNAs that were differentially enriched in oxidative phosphorylation pathways, including G-m0005, G-m0008/H-m0016, G-m0011/H-m0027 and G-m0015 miRNAs, that could target V-type H+-transporting ATPase subunit H and three subunits of cytochrome c oxidase (I, XV and XVII subunits) [63]. V-type H+ transporting ATPase subunit H involves the transport of protons across the mitochondrial membrane for ATP hydrolysis and pumping activity, whereas cytochrome c oxidase is also known as complex IV in the electron transport chain reaction. As previously mentioned, it is used to receive electrons from cytochrome c and react with oxygen atoms to form water molecules. Therefore, the downregulation of cytochrome c oxidase will over yield reactive oxygen species and cause oxidative damage to cellular processes. Other than that, Sun et al. (2016) revealed that two miRNAs (dpu-miR-745 and lgi-miR-745b) are differentially regulated in WSSV-infected shrimp *L. vannamei* which target ATP-binding cassette sub-family B member 10 (ABCB 10) in mitochondrial [64]. From NCBI and UniProt databases, ABCB 10 could catalyse to transport various molecules across extra- and intra-cellular membranes from mitochondrial matrix to cytoplasm in an ATP-dependent manner (https://www.ncbi.nlm.nih.gov/gene?Db=gene&Cmd=DetailsSearch&Term=56199, accessed on 27 February 2023) and (https://www.uniprot.org/uniprotkb/Q9NRK6/entry, accessed on 27 February 2023). In addition, Shekhar et al. (2019) also found two miRNAs (tca-miR-9e-5p and tcf-miR-9b-5p) targeted to NADH dehydrogenase subunit 4 that were significantly expressed in *L. vannamei* during WSSV-infection [65]. NADH dehydrogenase subunit 4 is a core unit of NADH dehydrogenase, which is also known as complex I used in the electron transport chain reaction (https://www.uniprot.org/uniprotkb/N0GSQ5/entry, accessed on 27 February 2023).

### 3.3. Apoptosis

Apoptosis is a process of cell-programmed cell death which promotes cellular balance and the elimination of unwanted cells without triggering inflammation in the organisms. Unlike necrosis, it involves accidental cell death caused by external factors that leads to the uncontrolled release of inflammatory contents in the organisms. In other words, apoptosis is an active and natural process that can be activated by a series of gene expressions and regulations in the cell. Recently, miRNAs have been studied that could induce cell apoptosis during pathogen infection. For instance, miR-92a could promote apoptosis in coelomocytes of sea cucumber *Apostichopus japonicus* during *V. splendidus* infection by targeting Bcl-2-associated X protein (Bax) [66]. They found that miR-92a could be a positive regulator of pro-apoptotic immune response by targeting Bax expression, and subsequently activating the other apoptosis-related genes and immune factors to resist the invasion of pathogens. In addition, miR-200 was found to suppress the expression of Toll interacting protein (Tollip) by binding to its 3′-UTR in *A. japonicus* in response to *V. splendidus* infection [67]. They also found that silencing of Tollip could increase apoptotic activity in primary coelomocytes after over-expression of miR-200. Apart from this, Qiao et al. (2022) investigated mRNA and miRNA expression profiles in orange-spotted grouper *Epinephelus coioides* during *V. parahaemolyticus* infection [68]. They revealed that miR-144-y, miR-378-y, novel-m0459-5p, and novel-m0440-5p miRNAs could regulate multiple target genes related to apoptosis. For instance, miR-144-y targets pro-apoptotic factor cytochrome c (cytc) and TP53 apoptosis effector (perp); miR-378-y regulates B cell leukemia/lymphoma 2 (BCL-2) family mitochondrial intrinsic anti-apoptotic protein and thrombospondin 1 (thbs1); novel-m0459-5p targets protein phosphatase 1D (ppm1d) and thbs1; novel-m0440-5p targets growth arrest and DNA damage-inducible 45 (gadd45) and caspase14. In their study, the expression of bcl2 was shown to be downregulated while the expression of cytc and caspase 14 were upregulated. This finding indicates apoptosis pathway may participate in antibacterial immunity in *E. coioides*. Moreover, Kaewkascholkul et al. (2016) characterized the miRNA expression in shrimp *P. monodon* against WSSV infection [69]. In their miRNA profiling, they found that downregulation of pmo-miR-9a-5p could activate the caspase 2 gene, which is a key enzyme used to activate the proteinase cascade apoptosis pathway. In addition to that, Soo et al. (2020) revealed that the apoptosis-related miRNA (dre-miR-107b) was downregulated in *V. parahaemolyticus*-infected *P. monodon* in 36 h post-infection which targets tumour protein p53-inducible protein 11 (pIG11) [70]. This indicates the target pIG11 enhances the cell apoptosis in the late stage of *Vp*AHPND-infected shrimps *P. monodon*. In short, the findings contributed to the knowledge of miRNA-mediated cell apoptosis against various diseases in aquaculture. 

## 4. MicroRNAs Enhance Replication of Pathogens in Host

**Figure 5:**
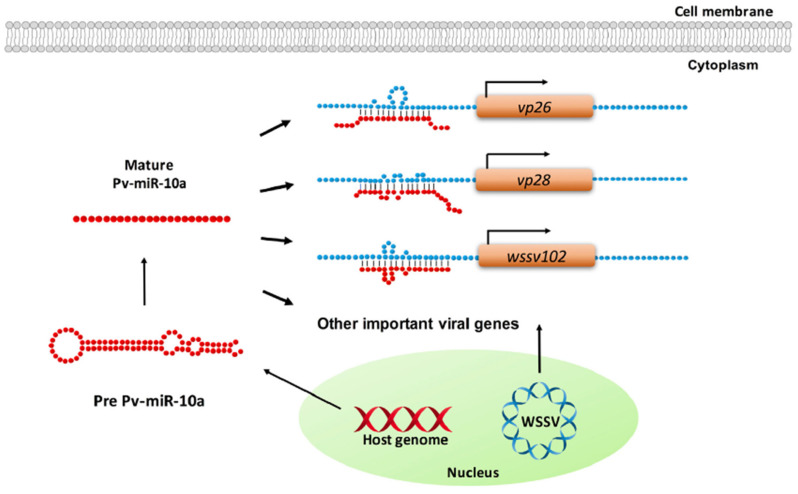
Schematic representation of how shrimp miR-10a is targeting 5′ untranslated region of WSSV genes to enhance viral replication. Reprinted from [75].

The abovementioned miRNAs have been shown to take part in immune responses during infection. However, miRNAs also regulate both viral or host gene expression to facilitate the replication of pathogens. Huang et al. (2016) found two miRNAs (miR-9041 and miR-9850) from shrimp *M. rosenbergii* that could enhance WSSV replication by suppressing STAT regulation and inhibiting downstream dynamin (Dmn) genes, namely, Dmn1, Dmn2, and Dmn3, which involves antiviral immunity [71]. Most miRNAs are suppressive as they can target the 3′-UTR of viral genes and inhibit their replications [72,73,74], but miRNAs can also be an enhancer of pathogens by binding to the 5′-UTR of viral genes. Huang et al. (2017) identified miR-10a from shrimp *L. vannamei* promoted WSSV replication by targeting 5′-UTR of WSSV genes, including *VP26*, *VP28*, and wsv102 [75] (Figure 5). In their study, they predicted that miR-10a might also target other WSSV structural and non-structural genes, such as *VP24*, *VP19*, WSSV DNA polymerase, *ie1*, *icp35*, *icp11,* and *rr2*, by using RNAhybrid software (http://bibiserv.techfak.uni-bielefeld.de/rnahybrid/, accessed on 27 February 2023).

Other than that, pmo-miR-315 was found to be significantly upregulated in the response to WSSV infection in shrimp *P. monodon* and its target was prophenoloxidase (proPO)-activating enzyme (PPAE3) [76]. PPAE3 is a novel serine proteinase that is activated by a series of cascades of serine proteinase in the proPO system. The proPO system is used to activate melanisation process, which is an important immune response and wound-healing mechanism, by the formation of melanin and preventing the loss of haemolymph and invasion of pathogens [77]. In a study by Jaree et al. (2018), they found that pmo-miR-315 reduced the transcription level of the PPAE3 gene, causing attenuation of proPO activity and facilitating the replication of WSSV [76]. In addition to that, one study has revealed that downregulation of miR-15b-5p in viral hemorrhagic septicemia virus (VHSV)-infected olive flounder *Paralichthys olivaceus* leads to upregulation of its target suppressor of cytokine signalling 6 (SOCS6) [78]. SOCS6 belongs to the SOCS family that suppresses JAK/STAT pathway to induce an antiviral response. However, other studies revealed that the inhibition of the viral gene could decrease the expression of SOCS6 and deactivate STAT1, which is a mediator for interferon responses. In the study by Lee’s group, they revealed that the downregulation of miR-15b-5p causes upregulation of SOCS6 and represses JAK/STAT pathway to evade the host immune response and increase VHSV replication. Interestingly, one study discovered that miR-100 could promote anti-vibrio through apoptosis, phagocytosis, and PO activity; on the other hand, it could also increase the progression of WSSV infection in shrimp *M. japonicus* [79]. However, its actual function in shrimp against *Vibrio* sp. and viral infection is still unknown.

As stated above, the host organisms encode different miRNAs to respond to the invasion of pathogens, but a lot of studies have discovered that viruses can produce viral-encoded miRNAs themselves to facilitate viral replication or evade the host immune responses [80,81,82,83,84]. In shrimp *M. japonicus*, WSSV-encoded miRNAs could target WSSV viral genes to promote a viral infection in which WSSV-miR-66 targets wsv094 and wsv177 genes while WSSV-miR-68 targets wsv248 and wsv309 genes [85]. In addition, another study revealed that WSSV-miR-22 regulated the host JAK/STAT-TEP1/TEP2 pathway and enhanced WSSV progression in shrimp *M. japonicus* [80]. Furthermore, Nantapojd et al. (2022) showed that WSSV-miR-9 was significantly expressed in the early stages of infection in shrimp *P. monodon*, hence they suggested WSSV-miR-9 facilitates WSSV infection [81]. However, this miRNA did not match crucial early genes of WSSV, such as ie1 and DNA polymerase, through the computational prediction method, and thus, the mechanism of WSSV-miR-9 to regulate the WSSV early gene expression is still unclear. On the other hand, He et al. (2020) identified 14 viral miRNAs in infectious spleen and kidney necrosis virus (ISKNV) infected cells through Illumina sequencing [86]. In their study, these 14 viral miRNAs, showed different effects in the infected cells, including the expression of major capsid protein (MCP) for viral replication purposes and affecting the changes of viral titers. For instance, inhibition of ISKNV-miR-c5, -c14, and -17-3p increased the expression of MCP but did not affect the virus titers; on the contrary, inhibition of ISKNV-miR-c4, -c9, and -c12 gave the opposite effects. Therefore, they suggested the virally encoded miRNAs could play varying roles in the viral life cycle which consists of attachment, penetration, uncoating, genome replication, expression, assembly, and release. In addition, one study investigated the function of Cyprinid herpesvirus 3-encoded miR-KT-635 in *Carassius auratus gibelio* caudal fin (GiCF) cell line [87]. They predicted that the target of this viral miRNA could be ORF23, which shares high homology with ribonucleotide reductase small subunit in the host, but its process of affecting the viral replication remains unclear. 

## 5. Current Applications of MicroRNAs

### 5.1. MicroRNAs Used as Biomarkers

By understanding the miRNA regulations that involve in the pathogenesis of infectious diseases, miRNAs could be used as biomarkers to detect diseases. Recently, several studies have focused on miRNAs, which can be used as a diagnostic tool for the identification of diseases, cancer, thermal tolerance effect, and reproductive and other stress effects in mammals and aquatic organisms [88,89,90,91,92,93,94,95,96,97,98]. For example, the report of El-Sebaey et al. (2020) described that Cfa-miR-122 and -21 could be used as serum biological markers to detect primary hepatitis in dogs as they were differentially expressed between the acute hepatitis group, chronic active hepatitis group, and healthy group [88]. In addition to that, five miRNAs (miR-124-3p, miR-34a-5p, miR-1-3p, miR-7-5p, and miR-99b-5p) were found in human cells that might target four hub genes (CXCL2, MMP9, SPP1, and SRC), which are related to inflammatory bowel disease (IBD) and hepatocellular carcinoma (HCC) [89]. Interestingly, miRNAs also have been used as sexual biomarkers to differentiate between male and pseudo-male fish *Cynoglossus semilaevis* [91]. Zhang’s group was the first to report determining exosomes and signature sexual miRNAs in seminal plasma from male and pseudo-males of *C. semilaevis*. They selected four miRNAs (dre-miR-141-3P, dre-miR-10d-5p, ssa-miR-27b-3p, and ssa-miR-23a-3p) as signature miRNAs which were significantly expressed in males and pseudo-males. Furthermore, in the review by Bhat et al. (2020), they compiled various miRNA expressions and their roles during different development stages of gonad and reproduction in both male and female teleost [99]. Therefore, the revealed miRNAs related to sexual reproduction could be biomarkers and targets to improve reproductive health and teleost species production. On the other hand, Nie et al. (2019) found differentially expressed miRNAs (miR-144-5p and miR-217-5p) between cold tolerant and cold sensitive in turbot *Scophthalmus maximus* [92]. Moreover, miRNAs can be applied as biomarkers to detect a wide range of infectious diseases. The miR-32 was first identified as cellular miRNA which is involved in RNA viral genome and reduced viral replication in human cells [100]. Currently, miRNAs also have been identified as biomarkers for various pathogens in humans, such as *Helicobacter pylori* [101], neonatal sepsis [102], nontuberculous mycobacterial pulmonary disease [103], HIV, HBV, HCV, HIV/HCV, HIV/HBV co-infection [104] and others. Through these, researchers investigated specific miRNAs to be the potential diagnostic tool for detecting infectious diseases in aquaculture. For instance, cid-miRn-115 and miR-142a-3p were identified in the kidney of susceptible grass carp and resistant grass carp infected with pathogenic *Aeromonas hydrophila*, which is a causative agent of hemorrhagic septicemia [96]. Moreover, various miRNAs were found as signatures in *V. harveyi*-infected *Cynoglossus semilaevis* fish [97,98]. Additionally, several studies have found that miR-144 could be a potential biomarker in teleost after different bacterial and viral infections [105,106,107]. In a study by Li et al. (2023), they found that miR-144 could inhibit the antibacterial immune response in flounder and promote the survival of bacterial *Edwardsiella tarda* [108]. Although miRNAs are evolutionarily conserved and have probably been used as universal biomarkers to detect pathogens among the species, they present challenges as stated by Samir and Pessler (2016) [109]. First and foremost, the presence of miRNA isomers might overlap with functional miRNAs and affect miRNAs’ stability and functionality [110]. In addition, miRNA biomarker studies might be biased due to the stability and miRNA yield extracted from biological samples, which requires a unique isolation protocol and centrifugation.

### 5.2. MicroRNAs Used as Therapeutic Agents

RNA interference (RNAi) is a form of post-transcriptional gene silencing (PTGS) that mediates the suppression of protein-coding genes by applying small-interfering RNA (siRNA) or miRNAs. The major difference between siRNA and miRNA is that siRNA is an exogenous double-stranded RNA that inhibits specific target mRNA, while miRNA is an endogenous non-coding RNA that inhibits or regulates multiple target mRNAs [111]. Thus, miRNAs have the potential to be therapeutic agents that rely on the gain and loss of function associated with miRNA expression in host organisms [112]. In mammals, miRNAs have been widely employed to control a variety of gene expressions and to reverse the normal cellular functional condition under diseases, either by miRNA inhibition or miRNA replacement. For instance, miR-29c was found to be a tumour suppressor that inhibits cell proliferation by targeting VEGFA expression in lung adenocarcinoma [113]; miR-221/222 acts as an anti-tumour agent to inhibit erythropoiesis through downregulation of c-Kit receptor [114]; inhibition of miR-21 might upregulate several tumour suppressor genes, the PI3K pathway and the TGF-β pathway, which could lead to suppressing prostate cancer [115]. Furthermore, miRNA therapies have been tested to treat ischemic heart diseases in different animal models, as stated by Kong et al. (2022) [116]. For example, inhibition of miR-132 rescued cardiac hypertrophy and heart failure in a rat model [117]. Other than that, miRNA therapy can also suppress infectious diseases such as HCV infection. The inhibition of miR-122 might block HCV infection and reduce viral plasma RNA levels from baseline [118]. Therefore, miRNA therapy has been widely applied in aquaculture. In the study by Dang et al. (2008), they delivered miR-MCPs and miR-HIRRV as pre-miRNA precursors into the hirame natural embryo (HINAE) fish cell line, leading to inhibition of viral infection by inducing interferon (IFN)-related pathways [119]. In addition, artificial miR-53Rs inhibited the viral protein RGV 53R in two fish cell lines (grass carp ovaries (GCO) and *epithelioma papulosum* cyprinid (EPC) cells) to avoid virion assembly [120]. Recently, shrimp miRNAs have been revealed to be an antiviral agent that inhibits WSSV infection. For instance, in the study by Cui et al. (2021), they delivered bacteria expressing miR-34 through shrimp fed into shrimp, resulting in the suppression of WSSV infection by targeting WSSV genes (wsv330 and wsv359) [121]. Interestingly, miR-34 was further tested in mice injected with breast cancer cells and fed cooked muscles of shrimp containing miR-34-expressing bacteria every two days. The results showed that miR-34 exhibited anti-tumour activity by targeting human genes (CCND1, CDK6, CCNE2, E2F3, FOSL1, and MET genes) in breast cancer mice. Thus, they speculated that miRNA could be used to control viral infection in shrimp, and the miRNA incorporated in shrimp muscle could be an effective strategy for controlling cancer in humans.

## 6. Current Trends of miRNA and Its Future Perspectives Used in Aquaculture

In this review, we have summarized the current status of miRNA expression profiling in aquaculture in response to various diseases for a better understanding of how miRNAs regulate infectious diseases and host immune responses. However, the mechanisms by which virally encoded miRNAs regulate replication and host immunity are still lacking, and this might limit the improvement of biomarker applications to detect pathogens and the development of therapeutic agents to treat certain diseases. Thus, bulk data are needed on miRNA expression profiling to compare infected and uninfected samples. To date, miRNA studies have mostly utilised quantitative PCR (qPCR) and next-generation sequencing (NGS) platforms to detect miRNAs. Despite that, these techniques have several limitations: low throughput, expensive, time-consuming, requirement of defined primers and probes for qPCR, complex sample preparation, and data analysis for NGS. Thereby, Tribolet et al. (2020) stated new detection platforms in the form of point-of-care (PoC) technologies to detect miRNAs, which are portable, reliable, rapid, low-cost, and user-friendly techniques [122]. For example, Zheng et al. (2018) designed a multiplex lateral flow miRNA biosensor to simultaneously detect miR-21, miR-155, and miR-210, which are related to cancers from human serum samples [123]; Shamsi et al. (2016) integrated electrochemiluminescence (ECL) technique into digital microfluidics (DMF) device to detect miR-143 in cancer cell lysates [124]. Other than that, in a study by Miti et al. (2020), they developed a miRNA biosensor based on localized surface plasmon resonance and hybridization chain reaction to detect miR-17, which is related to lung cancer and metastatic cancer [125]. In a study by Nehra et al. (2022), they designed an ultrasensitive electrochemical biosensor to detect miR-393a, which is an important miRNA linked to plant diseases [126].

In recent publications, various advanced miRNA biosensors have been developed to detect miRNA-related diseases. For instance, a dual DNA nanomachine-based homogeneous electrochemical biosensor was established to detect miR-141 as a model using a nicking enzyme-assisted cycling signal amplification technique [127]; Zhang’s group developed an ultrasensitive electrochemical biosensor based on antimonide quantum dot (AMQD)/ aromatic heterocyclic dyes and single-walled carbon nanotubes (SWCNTs) in 2023 for the simultaneous detection of miRNAs related to breast cancer (miR-21 and miR-155) [128]. Another technology was developed for the detection of miR-21 as well, which is a surface-enhanced Raman spectrum (SERS)-based miRNA biosensor that is based on porous metal-organic framework (MOFs) nanoparticles with subject-object recognition ability [129]. On the other hand, there is another technology called the Schottky diode to detect shrimp viruses by investigating DNA characteristics [130]. They proposed that this technology could also detect very small polynucleotides, such as siRNA and miRNA, by capturing their electrical signals and determining their electron charge transfer mechanism.

For now, no advanced miRNA detection technology has been developed for the aquaculture industry. Hence, this might be a breakthrough to invent miRNA biosensors to detect various miRNA-related diseases in aquaculture, especially for the detection of infectious diseases.

## Data Availability

Not applicable.
